# Cardiac Vigilance Beyond Hypertension With Lenvatinib in Thymic Carcinoma

**DOI:** 10.1111/1759-7714.70352

**Published:** 2026-07-13

**Authors:** Ashish Gupta, Amulya Gade, Ashish Subedi

**Affiliations:** ^1^ Internal Medicine Cape Fear Valley Medical Center Fayetteville North Carolina USA

Dear Editor,

1

We were very interested in the PECATI trial by Remon et al., which demonstrated Lenvatinib plus pembrolizumab efficacy in pre‐treated thymic malignancies, with report of grade 3 or worse cardiac dysfunction in one participant and myocarditis in another (2% each [1], but notably systemic echocardiographic monitoring was not part of the protocol). We present a case of severe delayed‐onset heart failure following introduction of Lenvatinib to ongoing chemotherapy upon disease progression, in a patient with prior cardiotoxic exposure.

A 72‐year‐old male patient with history of metastatic thymic carcinoma, status‐post thoracotomy, thymectomy, radiotherapy, and cisplatin‐based chemotherapy in the past. Patient remained on Gemcitabine and Capecitabine for roughly 5 years, with a stable but mildly reduced left‐ventricular ejection fraction (LVEF) of 45%–50%. Restaging computed tomography showed disease progression with new pulmonary and pleural‐based lesions, requiring addition of Lenvatinib 24 mg daily to the existing regimen. After 2 weeks, patient self‐discontinued lenvatinib secondary to new‐onset and intolerable bilateral otalgia, attributing to Lenvatinib's use. Three weeks later, the patient presented to the hospital with acute dyspnea and was noted to have heart failure with reduced ejection fraction of 10%–15%. Ischemic evaluation was negative for significant coronary artery disease.

Although this patient had a mildly reduced baseline LVEF to begin with, the cardiac dysfunction had remained stable for years, with acute decompensation noted after introduction of lenvatinib, supporting its temporal relationship to the patient's presentation. Lenvatinib, among all VEGFR tyrosine kinase inhibitors, has the strongest overall association with cardiovascular toxicity (reported Odds Ratio 6.815) with notable cardiac dysfunction signals included cardiac failure (reporting Odds Ratio 2.521) and Cardiomyopathy (reporting odds ratio 2.801) [2]. Beside our patient, Asakura et al. have published the only other description of lenvatinib‐induced heart failure in thymic carcinoma [3].

It is critical to highlight that before initiation of Lenvatinib, this patient had notably been exposed to cardiotoxic therapies over the years, including mediastinal radiation, cisplatin, gemcitabine and capecitabine. In a recent retrospective study of 410 patients with thymic neoplasms, radiation alone (HR 2.9) and pre‐existing cardiovascular comorbidities (HR 2.8) were each independently associated with cardiovascular events, with heart failure being the most common [4]. Patient's past cardiotoxic exposures with underlying mildly reduced LVEF reflects the possibility of accumulated subclinical injury from the extensive treatment history and already diminished cardiac reserve, which precipitated acute decompensation with brief lenvatinib exposure at a higher dose of 24 mg, than the 20 mg used in the PECATI trial.

We advocate for baseline and serial cardiac assessment including echocardiography and blood pressure monitoring for all patients, especially the ones with prior cardiotoxic exposures (including mediastinal radiation, platinum‐based chemotherapy), pre‐existing cardiomyopathy before initiation of Lenvatinib therapy.

## Author Contributions

**Ashish Gupta:** investigation, writing – review and editing, data curation, writing – original draft, validation. **Ashish Subedi:** conceptualization, supervision, project administration, writing – review and editing. **Amulya Gade:** investigation, writing – review and editing, validation.

## Funding

The authors have nothing to report.

## Consent

Written informed consent was obtained from the patient for the publication of this case report and any accompanying clinical information.

## Conflicts of Interest

The authors declare no conflicts of interest.

## Data Availability

The data that supports the findings of this study are available on request from the corresponding author. The patient data are not publicly available due to privacy or ethical restrictions.
